# Bundle branch re-entry ventricular tachycardia mimicking outflow-tract tachycardia: a case report

**DOI:** 10.1093/ehjcr/ytag520

**Published:** 2026-07-28

**Authors:** Khuong Dang Tran, Paula Victoria Catherine Brome, Dung The Bui, Nhut Minh Nguyen, Chin-Yu Lin

**Affiliations:** Heart Rhythm Unit, Cardiology Department, University Medical Center Ho Chi Minh City, 215 Hong Bang Street, Cho Lon Ward, Ho Chi Minh City 72723, Viet Nam; Heart Rhythm Center and Cardiovascular Center, Taipei Veterans General Hospital, No. 201, Section 2, Shipai Road, Beitou District, Taipei 112201, Taiwan; Heart Rhythm Unit, Cardiology Department, University Medical Center Ho Chi Minh City, 215 Hong Bang Street, Cho Lon Ward, Ho Chi Minh City 72723, Viet Nam; Heart Rhythm Unit, Cardiology Department, University Medical Center Ho Chi Minh City, 215 Hong Bang Street, Cho Lon Ward, Ho Chi Minh City 72723, Viet Nam; Heart Rhythm Center and Cardiovascular Center, Taipei Veterans General Hospital, No. 201, Section 2, Shipai Road, Beitou District, Taipei 112201, Taiwan; School of Medicine, College of Medicine, National Yang Ming Chiao Tung University, No. 155, Section 2, Linong Street, Beitou District, Taipei 112304, Taiwan

**Keywords:** Bundle branch re-entrant, Ventricular tachycardia, RVOT, Case report

## Abstract

**Background:**

Bundle branch re-entrant ventricular tachycardia (BBRVT) is a macro-re-entrant tachycardia involving the His–Purkinje system and is diagnosed by electrophysiologic study. However, atypical ventricular breakout may produce an electrocardiographic pattern mimicking ventricular tachycardia (VT) from right ventricular outflow tract (RVOT), resulting in misdiagnosis.

**Case summary:**

A 66-year-old woman with previous myocardial infarction presented with palpitation and sustained wide-complex tachycardia, left bundle branch block morphology, and an inferior axis, initially suggesting RVOT VT. During electrophysiological study, RVOT mapping failed to identify local ventricular electrograms preceding the QRS complex, and pace mapping was suboptimal. Detailed right ventricular mapping demonstrated that a discrete His potential consistently preceded every ventricular QRS complex during tachycardia. His–His interval changes preceded and predicted subsequent ventricular–ventricular interval changes. Entrainment from the right ventricular apex demonstrated a post-pacing interval minus tachycardia cycle length of 28 ms. These criteria supported BBRVT. Three-dimensional right ventricular activation mapping showed the earliest ventricular breakout at the sub-RVOT anteroseptal region, preceding activation at the right ventricular apex (the common breakout of BBRVT). This proximal septal breakout likely reflected faster conduction through the preserved septal branch than through the impaired distal right bundle branch (RBB). Radiofrequency ablation along the RBB region terminated the tachycardia. The patient remained free from recurrent VT during 6-month follow-up.

**Discussion:**

BBRVT may mimic RVOT VT when the ventricular breakout occurs at a sub-RVOT anteroseptal region. Not all ventricular arrhythmias with an RVOT-pattern ECG are focal and idiopathic.

Learning points

- Inferior-axis wide-complex tachycardia with LBBB morphology is not always RVOT VT; BBRVT can mimic an outflow-tract origin when the ventricular breakout is atypical.
- His–Purkinje involvement should be suspected when His potentials consistently precede QRS complexes and H–H interval changes precede V–V interval changes.

## Introduction

Bundle branch re-entrant ventricular tachycardia (BBRVT) is an uncommon macro-re-entrant ventricular tachycardia (VT) involving the His–Purkinje system. In the typical form, antegrade conduction over the right bundle branch (RBB) produces a left bundle branch block (LBBB) morphology, often with a superior axis, because ventricular breakout usually occurs from the distal RBB or right ventricular (RV) apical region.^1–3^ However, atypical breakout from the sub–right ventricular outflow tract (RVOT) anteroseptal region may generate an RVOT-like morphology and obscure the true re-entrant mechanism. We report a case of BBRVT presenting with LBBB morphology and inferior axis, initially mimicking RVOT tachycardia, in which detailed electrophysiological mapping revealed BBRVT with sub-RVOT anteroseptal breakout. This case emphasizes that not all RVOT-like ventricular arrhythmias are focal in origin and idiopathic.^4^

## Summary figure

**Table:** 

Time point	Event
> 1 year before presentation	Acute coronary syndrome treated with percutaneous coronary intervention and stenting of the mid-left anterior descending artery.
Day 0—emergency presentation	Palpitations; heart rate 203 bpm, hypotension; sustained wide-complex tachycardia with LBBB morphology and inferior axis. Synchronized cardioversion restored sinus rhythm with incomplete RBB block.
Day 0—initial workup	Normal troponin I; transthoracic echocardiography with preserved left ventricular function and no regional wall motion abnormality; coronary angiography showed a patent mid-left anterior descending stent.
Day 0—electrophysiology study	BBRVT diagnosed; radiofrequency ablation of the RBB terminated the tachycardia. Post-ablation electrogram showed complete RBB block.
1 month	Twelve-lead electrogram confirmed persistent complete RBB block.
2 months	7-day Holter monitoring showed no recurrent VT
3 months	Cardiac MRI demonstrated septal scar.
6 months	Asymptomatic, with no recurrence of VT.

## Case presentation

**Figure 1:**
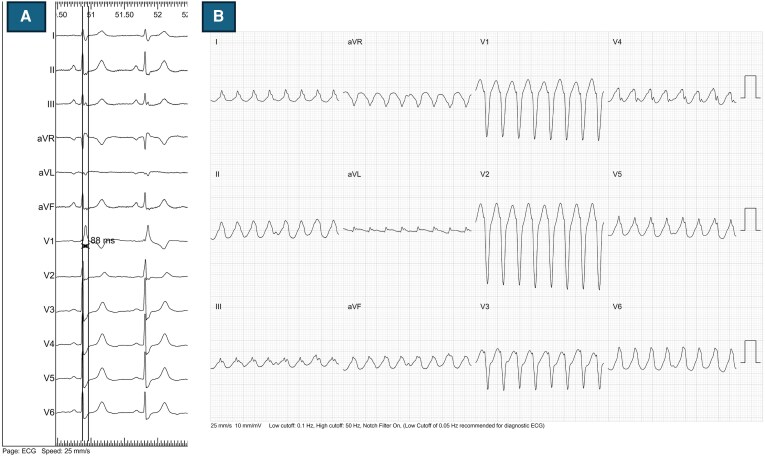
*(A)* Electrocardiograms of the patient’s baseline sinus rhythm (sinus cycle length 746 ms, PR interval 152 ms, QRS 88 ms, QT interval 418 ms, incomplete right bundle branch block). *(B)* Ventricular tachycardia with left bundle branch morphology and inferior axis (tachycardia cycle length 260 ms). ms = milliseconds.

We present a case of a 66-year-old female who consulted in the emergency room for palpitations lasting 1 h. Past medical history revealed previous acute coronary syndrome status post stenting in the left anterior descending artery (LAD) and maintenance medications of aspirin, clopidogrel, pravastatin, bisoprolol, and valsartan. On evaluation, the patient had a heart rate of 203 bpm and hypotension (95/65 mmHg), without evidence of pulmonary oedema or other end-organ hypoperfusion. The electrocardiogram (ECG) showed VT: cycle length of 260 milliseconds (ms), QRS duration of 120 ms, LBBB morphology, inferior axis, V4 transition, and ventriculoatrial dissociation (*Figure 1B*). Synchronized cardioversion with 100 J restored sinus rhythm with incomplete right bundle branch block (RBBB) morphology (QRS duration 88 ms). Initial workup showed normal troponin I, and coronary angiogram showed a patent stent in the mid-LAD. Left ventriculogram and echocardiography showed preserved left ventricular (LV) systolic function without regional wall motion abnormality. Repeat coronary angiography was performed in order to exclude an acute ischaemic trigger for the tachycardia. With an initial impression of inferior axis VT, possibly arising from the lower RVOT given the relatively modest R-wave amplitude in the inferior leads, and considering the patient’s prior myocardial infarction, a scar-related re-entrant mechanism was also suspected. Therefore, we proceeded with electrophysiologic study and possible catheter ablation.^5^

**Figure 2:**
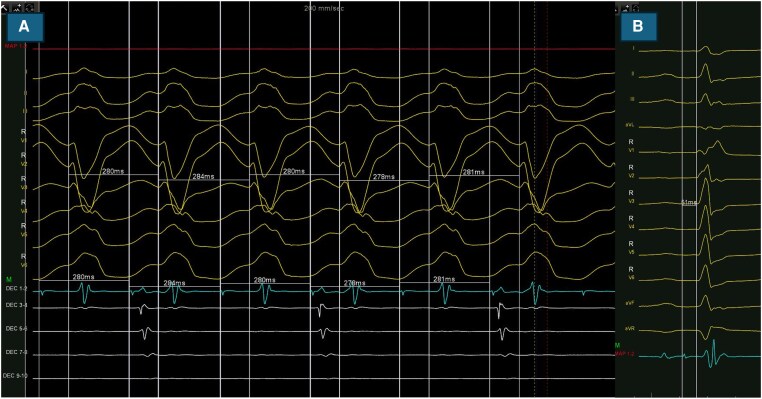
*(A)* A discrete His potential was consistently recorded before each QRS complex on DEC 1–2 (DECANAV mapping catheter at His region). Importantly, beat-to-beat changes in the His–His interval preceded and predicted the subsequent ventricular–ventricular interval. *(B)* HV interval 51 ms during sinus rhythm. HV = His-ventricular; ms = milliseconds.

**Figure 3:**
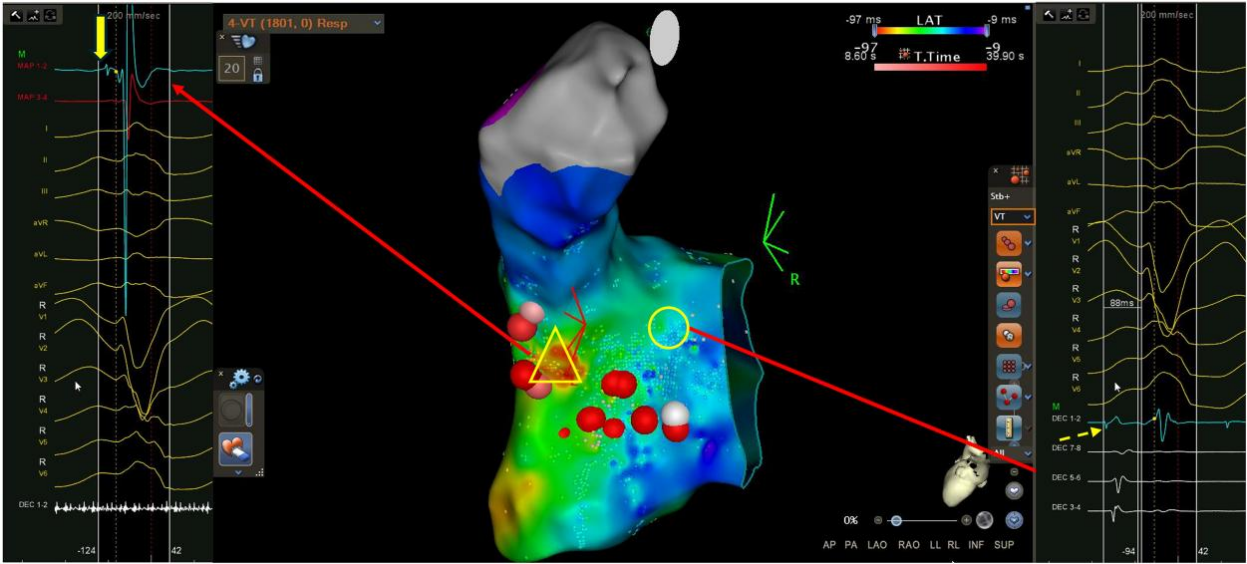
Right ventricular activation map during bundle branch re-entrant ventricular tachycardia. The red-coloured region (yellow triangle) located at the right ventricular sub-RVOT anteroseptum represents the earliest ventricular breakout site, which preceded activation at the right ventricular apical region. Red ablation tags indicate radiofrequency applications delivered along the presumed right bundle branch course from the mid-septal region towards the right ventricular apex. The solid yellow arrow indicates the right bundle branch potential, whereas the dashed yellow arrow indicates the His bundle potential. RVOT = right ventricular outflow tract.

**Figure 4:**
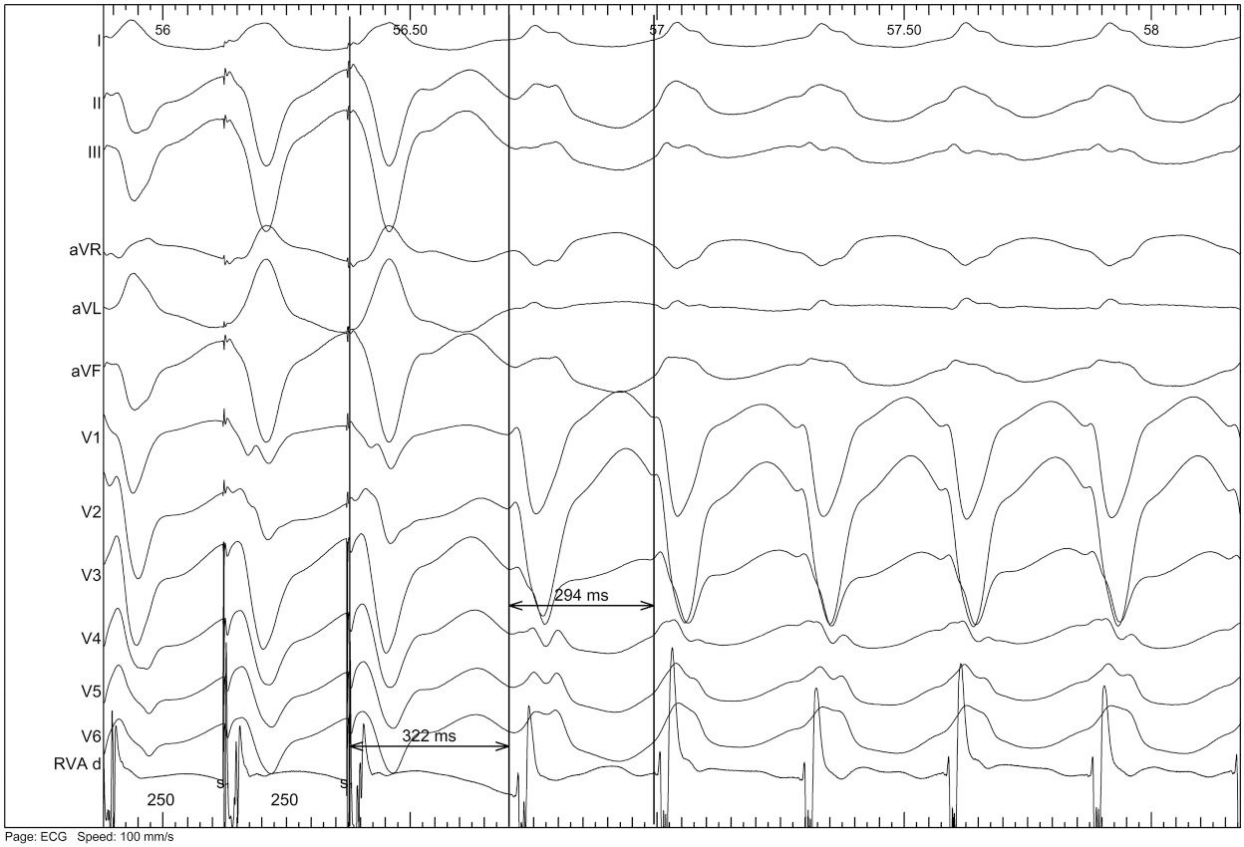
Right ventricular apical entrainment showing post-pacing interval minus tachycardia cycle length of 28 ms, indicating that it is within the circuit. ms = milliseconds.

**Figure 5:**
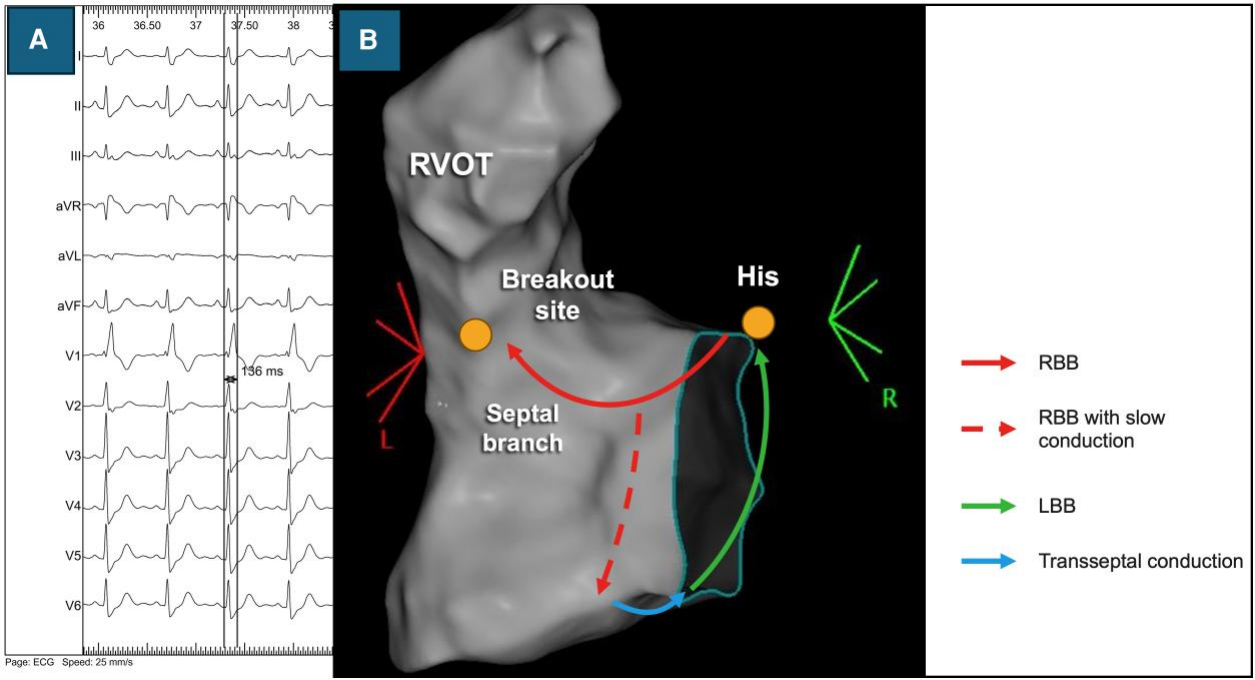
*(A)* Twelve-lead electrocardiogram recorded after right bundle branch (RBB) ablation, showing complete right bundle branch block (RBBB) with prolongation of the QRS duration to 136 ms. *(B)* Schematic illustration of the proposed bundle branch re-entry circuit on the three-dimensional right ventricular geometry: antegrade conduction down the RBB (solid red arrow) with ventricular breakout at the sub–right ventricular outflow tract (RVOT) antero-septal region via a septal branch, slow distal-RBB conduction towards the right ventricular apex (dashed red arrow), transseptal conduction (blue arrow), and retrograde conduction up the left bundle branch (LBB, green arrow) returning to the His bundle.

Baseline rhythm during the study was sinus with a cycle length of 746 ms, AH interval of 96 ms, HV interval of 51 ms, and QRS duration of 88 ms (*Figure 1A*, *Figure 2B*). VT was induced with RV burst pacing. The induced VT showed the same morphology as the clinical tachycardia (*Figure 1B*). Placing the decapolar mapping catheter (DECANAV, Biosense Webster, Inc., Irvine, CA, USA) in the RV septum during tachycardia, a His signal was noted consistently before the V signal with an HV interval of 88 ms (*Figure 3*); H–H interval changes preceded V–V interval changes (*Figure 2A*). Three-dimensional right ventricular activation mapping showed the earliest ventricular breakout at the sub-RVOT anteroseptal region (*Figure 3*). RBB potentials were also noted before the V signals in this area with RB-V interval of 18 ms (*Figure 3*). We proceeded to perform maneuvers to rule out other differential diagnoses. Pace-mapping was done using a decapolar. However, best pace map only revealed 85.4% correlation from the lower RVOT (see Supplementary material online, *Figure S2*). The earliest ventricular activation was recorded at this site, preceding the surface QRS onset by 16 ms. The unipolar electrogram showed a small r wave with a large S wave (rS configuration). Atrial extrastimulus testing did not demonstrate a distinct AH interval jump-up, and adenosine administered during tachycardia showed no change, making atrioventricular re-entrant tachycardia less likely. Entrainment from the RV septal apex also showed a short post-pacing interval (PPI) minus tachycardia cycle length (TCL) (28 ms), indicating that the RV septal apex area was within the circuit (*Figure 4*). With the impression of BBRVT, we proceeded to ablate the RBB during VT using a Thermocool ablation catheter (Biosense Webster, Inc., Irvine, CA, USA) with a power of 30 watts for 60 s per radiofrequency application. The tachycardia terminated within 1.2 s of energy delivery and additional consolidation lesions were done in the same area (*Figure 3*) to achieve complete RBBB. Inducibility testing with isoproterenol 4 mcg/min after ablation could not induce any tachycardia. Post-ablation sinus rhythm ECG showed complete RBBB with a longer QRS duration of 136 ms (*Figure 5A*). The patient tolerated the procedure well. A 12-lead ECG obtained 1 month after the procedure confirmed persistent complete RBBB (see Supplementary material online, *Figure S1*), and a 7-day Holter monitor performed 2 months after the procedure recorded no recurrent VT; the patient remained asymptomatic and free from recurrent VT throughout 6 months of follow-up. Cardiac magnetic resonance (CMR) was done 3 months after the procedure showing some late gadolinium enhancement (LGE) in the RV septum (see Supplementary material online, *Figure S3*).

## Discussion

The diagnosis of BBRVT was confirmed by several electrophysiological hallmarks: LBBB morphology, a His-bundle potential preceding each ventricular signal, beat-to-beat changes in the H-H interval preceding and predicting subsequent V-V interval changes, and termination of the tachycardia solely through RBB ablation. Furthermore, the HV interval during tachycardia was 88 ms, falling within the 30–135 ms range characteristic of BBRVT, and the PPI–TCL was 28 ms during entrainment at the RV apex.^1,2^ The tachycardia utilized antegrade conduction through the RBB. In our case, retrograde conduction through the LBB was inferred from the diagnosis of BBRVT rather than directly demonstrated, because left-sided mapping was not performed. This represents a limitation of our case. Importantly, in a patient with ischaemic cardiomyopathy, re-entrant VT should remain the leading diagnostic consideration. Interfascicular re-entry was unlikely because of the non-RBBB morphology. Focal RVOT VT was excluded by reproducible RV apical entrainment, supporting re-entry. AVRT, including nodofascicular pathway-mediated AVRT, was unlikely because of AV dissociation and the short PPI–TCL, as AVRT with nodofascicular pathway would usually be expected to show a longer PPI–TCL from the RV apex entrainment. Scar-related re-entry with bystander His–Purkinje activation was unlikely because H–H drove V–V intervals.

Regarding inferior axis and LBBB morphology, we theorize that the scar at the septum as evidenced by the MRI performed later (see Supplementary material online, *Figure S3*) could have disrupted the conduction of the RBB, resulting in an incomplete block during sinus rhythm before the procedure. This pre-existing RBB disease may predispose the patient to present with an unusual morphology of a BBRVT. We hypothesize that the ‘RVOT-like’ morphology resulted from a proximal breakthrough from the RBB system into the RV myocardium, possibly at the sub-RVOT anteroseptal region, rather than a distal exit due to preserved and faster septal Purkinje conduction to this region compared to impaired and slower RBB conduction to the RV apex. RV activation mapping supported this hypothesis by demonstrating the earliest RV myocardial activation at the sub-RVOT anteroseptal region, earlier than that recorded at the RV apex (*Figure 3*). However, this site was only interpreted as the ventricular breakout. Meanwhile, the critical re-entrant isthmus included His, RBB, RV apex, and LBB (*Figure 5B*). This was supported by entrainment from the RV apex yielding a PPI–TCL < 30 ms, indicating that the apical region was closely linked to, or part of, the re-entrant circuit (*Figure 4*). This finding is in line with RBB anatomy. Because the RBB normally courses along the ventricular septum towards the moderator band and gives rise to septal Purkinje branches along this pathway.^6^ In our case, we suspect that impulses are conducted and break out at the high-to-mid anteroseptal RV septum via the septal branch of RBB, immediately below the RVOT, providing a plausible explanation for the inferior-axis morphology.

CMR performed 3 months after the procedure showed transmural-appearing septal LGE, possibly reflecting a mixed aetiology (see Supplementary material online, *Figure S3*). The LV subendocardial-to-intramural component is more consistent with prior infarct-related scar, whereas the RV endocardial component may be partly related to ablation-induced injury. Because CMR was performed 3 months after ablation, the relative contribution of each process cannot be determined with certainty. Although LV systolic function was preserved, the septal myocardial scar identified on CMR—partly related to the patient’s prior myocardial infarction—may have provided the substrate for His–Purkinje conduction delay and the development of BBRVT. This is consistent with prior observations that BBRVT may occur in the setting of His–Purkinje conduction disturbances, even when overt structural heart disease is absent.^7^

## Conclusion

BBRVT typically occurs in patients with underlying His–Purkinje disease, and because this disease could alter conduction along the RBBB, the ventricular breakout of LBBB-morphology BBRVT is not always at the RV apex—it may occur at other sites along the RBB, including the sub-RVOT antero-septal region, producing an ECG pattern that mimics RVOT ventricular arrhythmias. Conversely, not every RVOT-pattern ECG VT is idiopathic or focal in origin.

## Supplementary Material

ytag520_Supplementary_Data

## Data Availability

The data underlying this article will be shared on reasonable request to the corresponding author.

## Lead author biography

Dr Khuong Dang Tran is a cardiac electrophysiologist with 5 years of experience in the diagnosis and interventional management of cardiac arrhythmias. His clinical practice focuses on electrophysiology studies, catheter ablation, pacemaker implantation, and implantable cardioverter-defibrillator therapy. He is currently affiliated with University Medical Center Ho Chi Minh City, one of the largest tertiary referral and central government hospitals in southern Vietnam.

## Notes

**Consent:** The authors confirm that written consent for submission and publication of this case report, including images and associated text, has been obtained from the patient, in line with COPE guidance.

## Patient perspective

Not available.
