# Supplementary material for: Bundle branch re-entry ventricular tachycardia mimicking outflow-tract tachycardia: a case report
Source: Eur Heart J Case Rep. 2026 Jul 28;10(7):ytag520. doi: 10.1093/ehjcr/ytag520 (PMC13412384; doi:10.1093/ehjcr/ytag520)
Supplement: ytag520_Supplementary_Data [file ytag520_supplementary_data.zip › Supplemental figure legends.docx]

**SUPPLEMENTARY FIGURE**

**Supplementary Figure 1.** Twelve-lead electrocardiogram recorded one month after the procedure, demonstrating persistent complete right bundle branch block.

**Alt text:** A 12-lead electrocardiogram recorded one month after the procedure showing a persistent complete right bundle branch block pattern.

**Supplementary Figure 2.** Pace mapping in the right ventricle. The site of best pace-map correlation (85.4%) was located in the lower right ventricular outflow tract, corresponding to the earliest ventricular breakout site identified on the activation map. The right-hand panel shows the 12-lead pace-map correlation between the paced morphology and the clinical ventricular tachycardia.

**Alt text:** A right ventricular electroanatomic map marking the best pace-map site in the lower right ventricular outflow tract, shown beside a side-by-side 12-lead comparison of the paced QRS morphology and the clinical tachycardia, with an 85.4% correlation.

**Supplementary Figure 3.** Cardiovascular magnetic resonance (late gadolinium enhancement) image of the interventricular septum. The yellow arrow indicates transmural septal enhancement, consistent with the patient's prior myocardial infarction; the red arrow indicates enhancement along the right ventricular endocardial aspect of the septum, likely related to radiofrequency ablation injury.

**Alt text:** A cardiac magnetic resonance image of the interventricular septum showing bright late-gadolinium-enhancement signal. A yellow arrow points to transmural enhancement across the septum, and a red arrow points to enhancement along the right ventricular side of the septum.
