# Supplementary figures and images for: Bundle branch re-entry ventricular tachycardia mimicking outflow-tract tachycardia: a case report
Source: Eur Heart J Case Rep. 2026 Jul 28;10(7):ytag520. doi: 10.1093/ehjcr/ytag520 (PMC13412384; doi:10.1093/ehjcr/ytag520)

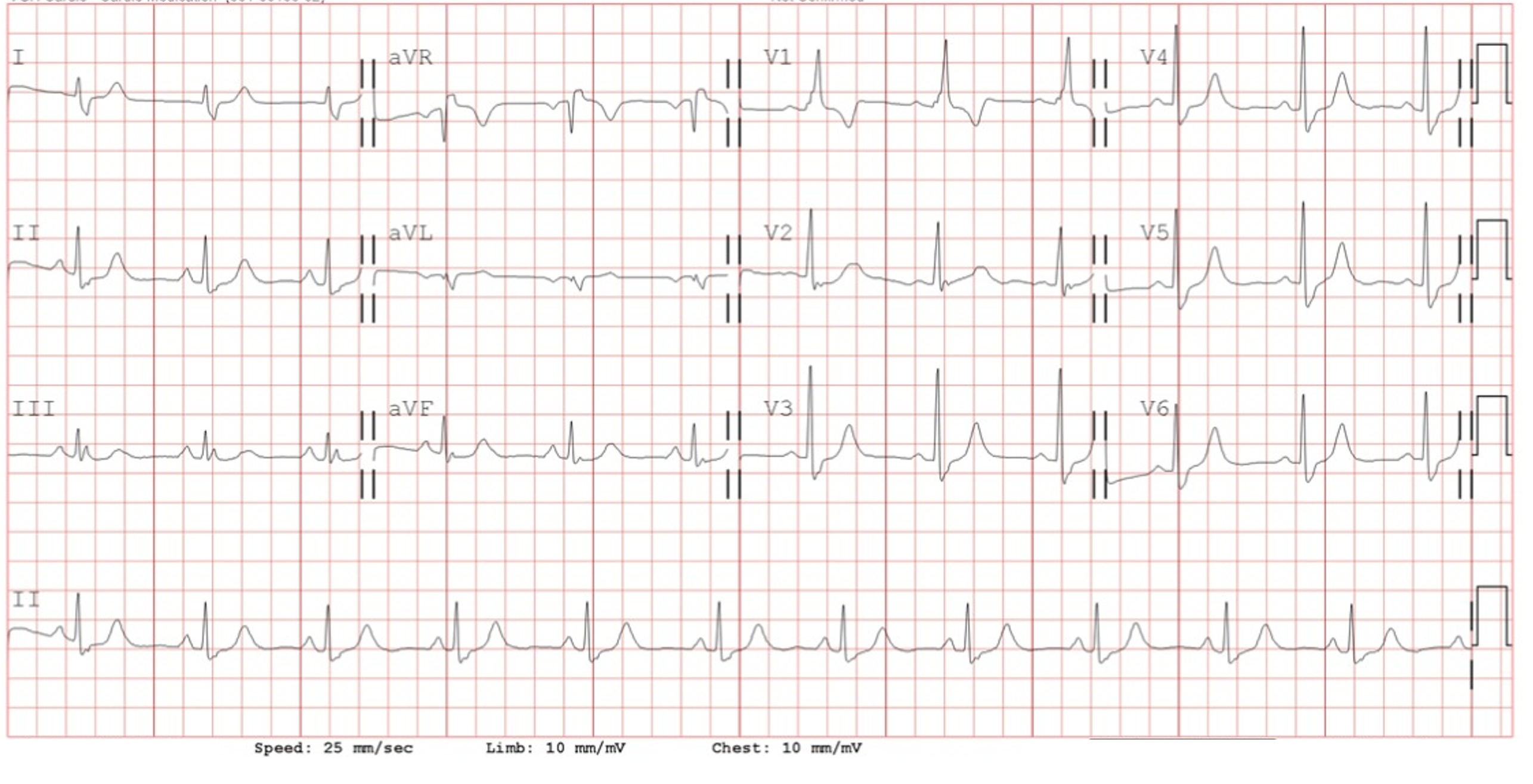

Supplement: ytag520_Supplementary_Data [file ytag520_supplementary_data.zip › Supplementary Figure 1.png]

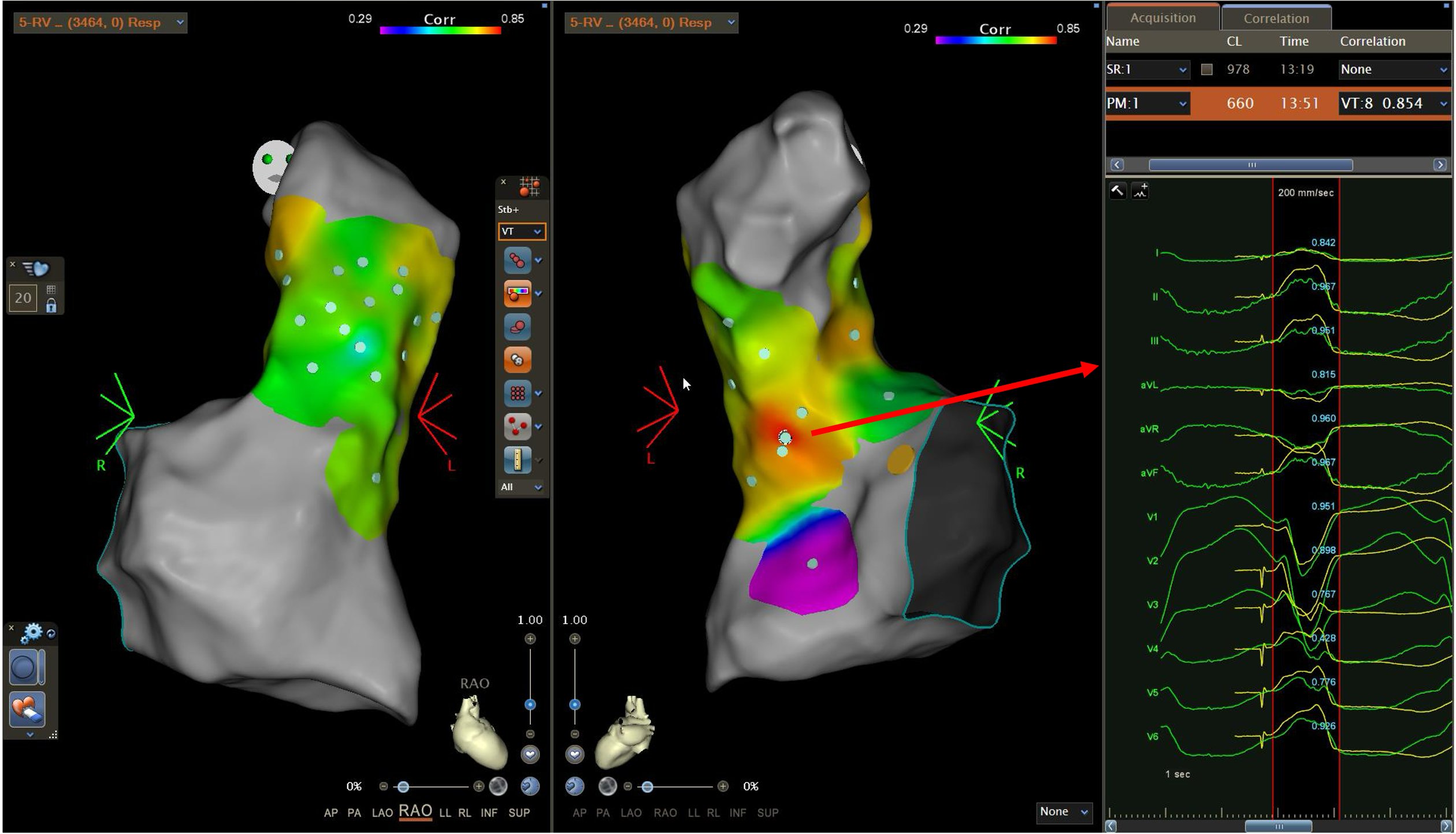

Supplement: ytag520_Supplementary_Data [file ytag520_supplementary_data.zip › Supplementary Figure 2.png]

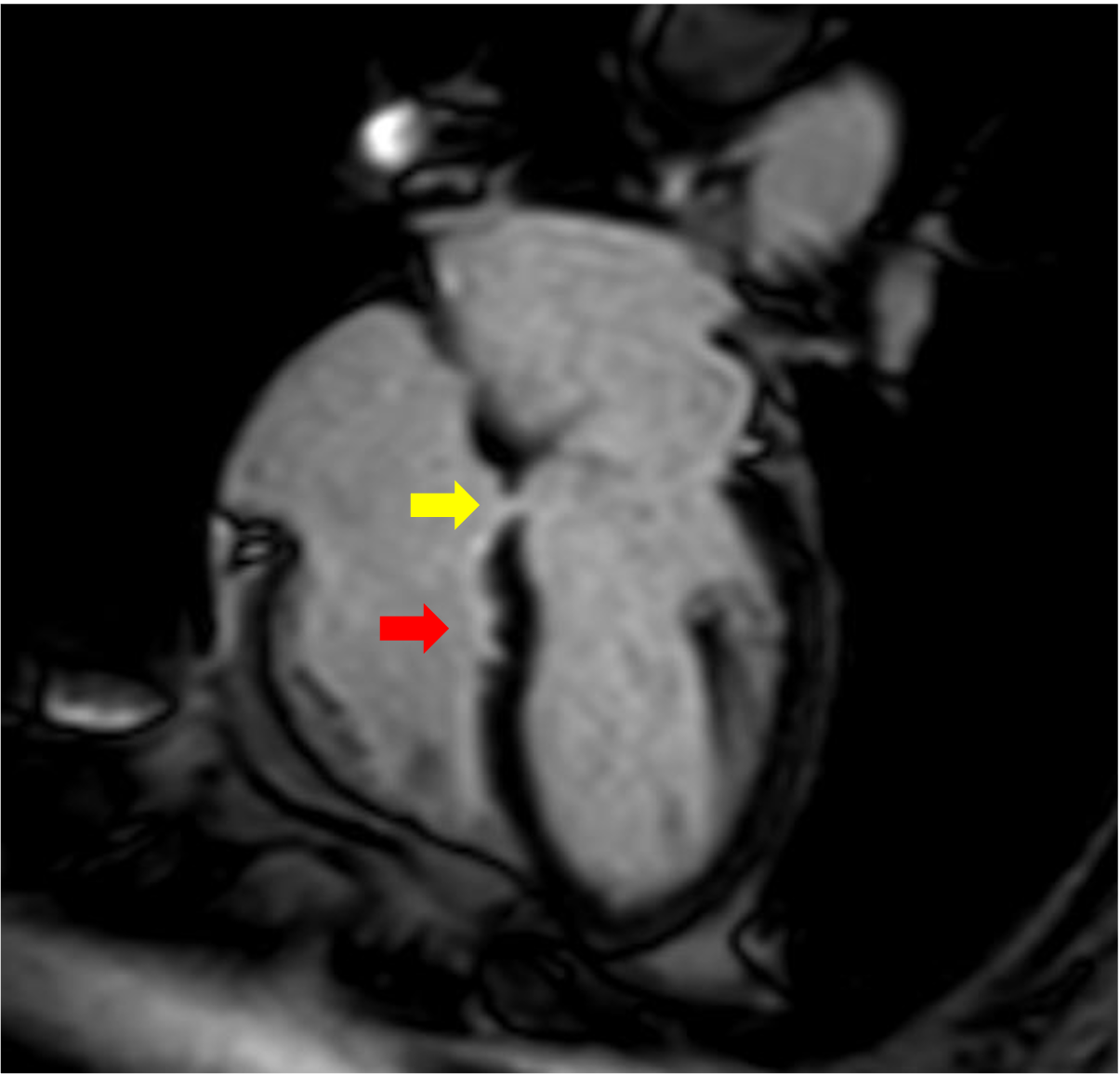

Supplement: ytag520_Supplementary_Data [file ytag520_supplementary_data.zip › Supplementary figure 3.png]
